# Ultrastructural Changes in Clinical and Microbiota Isolates of *Klebsiella pneumoniae* Carriers of Genes *bla*_SHV_, *bla*_TEM_, *bla*_CTX-M_, or *bla*_KPC_ When Subject to *β*-Lactam Antibiotics

**DOI:** 10.1155/2015/572128

**Published:** 2015-09-28

**Authors:** Dyana Leal Veras, Ana Catarina de Souza Lopes, Grasielle Vaz da Silva, Gabriel Gazzoni Araújo Gonçalves, Catarina Fernandes de Freitas, Fernanda Cristina Gomes de Lima, Maria Amélia Vieira Maciel, Ana Paula Sampaio Feitosa, Luiz Carlos Alves, Fábio André Brayner

**Affiliations:** ^1^Setor de Microscopia Eletrônica, Laboratório de Imunopatologia Keizo Asami (LIKA), Universidade Federal de Pernambuco (UFPE), Avenida Professor Moraes Rego, s/n, Cidade Universitária, 50670-901 Recife, PE, Brazil; ^2^Departamento de Parasitologia, Centro de Pesquisas Aggeu Magalhães (CPqAM)-Fiocruz, Avenida Professor Moraes Rego, s/n, Caixa Postal 7472, Cidade Universitária, 50670-420 Recife, PE, Brazil; ^3^Departamento de Medicina Tropical, Universidade Federal de Pernambuco (UFPE), Avenida Professor Moraes Rego, s/n, Cidade Universitária, 50670-901 Recife, PE, Brazil; ^4^Departamento de Enfermagem, Faculdade de Ciências Humanas de Olinda (FACHO), Rod PE-015, Jatobá, 53060-775 Olinda, PE, Brazil; ^5^Instituto de Ciências Biológicas, Universidade de Pernambuco (UPE), Rua Arnobio Marques 310, Santo Amaro, 50100-130 Recife, PE, Brazil

## Abstract

The aim of this study was to characterize the ultrastructural effects caused by *β*-lactam antibiotics in *Klebsiella pneumoniae* isolates. Three *K. pneumoniae* clinical isolates were selected for the study with resistance profiles for third-generation cephalosporins, aztreonam, and/or imipenem and with different resistance genes for extended-spectrum *β*-lactamases (ESBL) or *Klebsiella pneumoniae* carbapenemase (KPC). Two *K. pneumoniae* isolates obtained from the microbiota, which were both resistant to amoxicillin and ampicillin, were also analyzed. In accordance with the susceptibility profile, the clinical isolates were subjected to subminimum inhibitory concentrations (sub-MICs) of cefotaxime, ceftazidime, aztreonam, and imipenem and the isolates from the microbiota to ampicillin and amoxicillin, for analysis by means of scanning and transmission electron microscopy. The *K. pneumoniae* isolates showed different morphological and ultrastructural changes after subjection to *β*-lactams tested at different concentrations, such as cell filamentation, loss of cytoplasmic material, and deformation of dividing septa. Our results demonstrate that *K. pneumoniae* isolates harboring different genes that encode for *β*-lactamases show cell alterations when subjected to different *β*-lactam antibiotics, thus suggesting that they possess residual activity *in vitro*, despite the phenotypic resistance presented in the isolates analyzed.

## 1. Introduction

*Klebsiella pneumoniae* is one of the most common Gram-negative multidrug-resistant (MDR) organisms found worldwide, and it is responsible for high morbidity and mortality both in hospitals and in other healthcare units [1].

*β*-Lactam antibiotics are the most commonly prescribed antibacterial agents because of the high efficiency of their mechanism of action and low toxicity [2]. Penicillin-binding proteins are the lethal targets of *β*-lactams in sensitive bacteria and their inhibition leads to degradation of the bacterial cell wall and eventual cell lysis [3]. Several studies have reported different bacterial morphological changes caused by subminimum inhibitory concentrations (sub-MICs) of third-generation cephalosporins and carbapenems in sensitive isolates of several bacterial species [4–6]. However, there is a lack of studies using resistant isolates.

In pathogenic bacteria, *β*-lactamase production is the most common and important mechanism of resistance to *β*-lactam antibiotics [2]. The classical *β*-lactamases are enzymes capable of inactivating penicillins and narrow-spectrum cephalosporins before reaching their target [7]. Most* K. pneumoniae* isolates carry the gene for chromosomal class A SHV-1 *β*-lactamase. This has limited activity against ampicillin and does not hydrolyze extended-spectrum *β*-lactam antibiotics [8]. The presence of this gene has been previously studied in* K. pneumoniae* isolates originating from the microbiota of individuals without bacterial infection [9].

The introduction into clinical practice of oxyiminocephalosporins for treating infections due to Gram-negative bacteria was soon followed by the appearance of extended-spectrum *β*-lactamases (ESBLs) derived from the classical *β*-lactamases SHV-1, TEM-1, and TEM-2. These conferred resistance to third-generation cephalosporins and monobactams such as aztreonam [2]. More than 180 variants of the gene *bla*_SHV_ and more than 210 of the gene *bla*_TEM_ have already been reported [10]. Another group of ESBLs, which has a high profile of hydrolysis in relation to cefotaxime and is less than 40% identical to the SHV and TEM enzymes, is the group of CTX-M enzymes. These are widely found in different species of the family Enterobacteriaceae, including* K. pneumoniae* [11].

Treatment of infections due to* K. pneumoniae* isolates producing ESBLs is limited to the use of a few available antibiotics. Carbapenems are often the last line of effective treatment against these infections [12]. However, production of the class A carbapenemase* Klebsiella pneumoniae* carbapenemase (KPC), which is an extended-spectrum *β*-lactamase, has been reported to be the primary form of carbapenem resistance in this bacterial species. Fifteen variants with dissemination in different parts of the world have been reported [10].

Production of these *β*-lactamases by clinical isolates of* K. pneumoniae* has been a major problem in many countries, and this has hampered the therapeutic options available for the treatment of bacterial infections caused by this species.

The increasing prevalence of multidrug resistance is leading towards the threat that a postantibiotic era may be about to begin, characterized by decreased effectiveness of common antibiotics and routine application of complementary therapeutic approaches for treating bacterial infections [13]. Therefore, new studies need to be developed, in which old and discarded antibiotics should be reinvestigated and reused. Even rejected antibiotics possibly should be used when necessary [14].

In this regard, some studies have tried to demonstrate the* in vitro* and* in vivo* action of therapeutic combinations of *β*-lactams and aminoglycosides in MDR* K. pneumoniae* isolates, aiming to obtain new options for treating infections due to this bacterial species [15]. However, no study has demonstrated any alterations induced by different *β*-lactam antibiotics in the bacterial cell structure of MDR* K. pneumoniae* isolates.

*K. pneumoniae* isolates presenting resistance to cefotaxime when subjected to sub-MICs of this antibiotic were shown to cause damage to cell surfaces. Filamentation through a dose-dependent adaptive process was observed, caused by the stressful environment that was induced by the presence of the antibiotic, thereby contributing towards the therapeutic effects [16].

Unfortunately, knowledge about the effects caused by *β*-lactam antibiotics on the bacterial cell structure of MDR* K. pneumoniae* isolates is still scarce. These isolates carry important resistance genes, such as *bla*_SHV_, *bla*_TEM_, *bla*_CTX-M_, and *bla*_KPC_. Likewise, little is known about the effect of these antibiotics on isolates from the microbiota of individuals without bacterial infection. Therefore, the present study aimed to characterize the morphological and ultrastructural effects on pathogenic and nonpathogenic* K. pneumoniae* isolates that carry genes encoding the classical *β*-lactamases ESBL and KPC, caused by *β*-lactam antibiotics that are widely used in clinical medicine.

## 2. Material and Methods

### 2.1. Bacterial Isolates

For this study, five* K. pneumoniae* isolates from Recife, PE, Brazil, were selected (Tables 1 and 2). The K21.1-F and K58.1-F isolates were obtained from the fecal microbiota of children who did not have bacterial infection. These isolates showed resistance or intermediate resistance to ampicillin and/or amoxicillin but lacked the *bla*_SHV_ gene (Table 1) [9]. Three other* K. pneumoniae* clinical isolates obtained from public hospitals in Recife were used: K3C, K16R, and K652. The susceptibility profile and presence of the genes *bla*_SHV_ and *bla*_CTX-M_ had been determined in previous studies on the K3C and K16R isolates [9, 17], which showed resistance to cefotaxime, ceftazidime, and aztreonam, according to the interpretive criteria of the Clinical and Laboratory Standards Institute (CLSI) of 2013 [18].

The K3C isolate possesses the *bla*_SHV-11_ gene and the K16R isolate has the *bla*_SHV-1_ and *bla*_CTX-M2_ genes [9, 17]. The K652 isolate, which was studied for the first time in the present work, was obtained by means of surveillance testing on rectal swabs from individuals without clinical symptoms of bacterial infection who were admitted to a public hospital in 2011. This isolate was identified and its susceptibility profile in relation to different antimicrobials was determined using the VITEK 2 automated system (BioMérieux). It was manually confirmed in relation to cefotaxime, ceftazidime, and imipenem (Sigma-Aldrich) by means of broth macrodilution testing, in accordance with the 2013 CLSI recommendations [18].

### 2.2. DNA and PCR Extraction

The genomic DNA of* K. pneumoniae* isolates was extracted using the Wizard Genomic DNA Purification Kit (Promega), in accordance with the manufacturer's instructions. The presence of the *bla*_TEM_ gene was investigated by means of PCR in the macrobiota isolates and in the three clinical isolates using the T1 primer 5′-ATAAAATTCTTGAAGACGAAA-3′ and the T2 primer 5′-GACAGTTACCAATGCTTAATC-3′ [19]. The K652 isolate was also evaluated by means of PCR for the presence of the *bla*_SHV_ and *bla*_KPC_ genes using the primers 5′-GGTTATGCGTTATATTCGCC-3′ and 5′-TTAGCGTTGCCAGTGCTC-3 [20] and using KPC1F: 5′-GCTACACCTAGCTCCACCTTC-3′ and KPC1R: 5′-ACAGTGGTTGGTAATCCATGC-3′ [21], respectively.

### 2.3. DNA Sequencing

Sequencing of the PCR products from the *bla*_TEM_ and *bla*_KPC_ genes was performed using the Applied Biosystems/Hitachi automated sequencer, after purification of the PCR amplicons using the PureLink Micro Kit (Invitrogen), in accordance with the manufacturer's instructions. The *bla*_TEM_ gene was sequenced in the K3C and K16R isolates because of phenotypic resistance that was presented in relation to the third-generation cephalosporins that were tested. Only the *bla*_KPC_ gene was sequenced in K652 isolate, due to phenotypic resistance to carbapenems that was presented. The primers were the same as for PCR, plus TEMup21: 5′-TCCCTTTTTTGCGGCATTTTGC-3′ and TEMdwn280: 5′-CAGTGAGGCACCTATCTC-3′ [22], for the *bla*_TEM_ gene, and KPC F: 5′-GAGCTGAACTCCGCCATC-3′ and KPC R: 5′-TATTTTTCCGAGATGGGTGAC-3′ [23], for the *bla*_KPC_ gene.

The analyses on the DNA sequence and the multiple alignments were performed using the DNAstar software and BLAST [24]. The sequences for the *bla*_TEM-1_, *bla*_TEM-15_, and *bla*_KPC-2_ genes were deposited in the GenBank database under the access numbers KF906436, KF906435, and KF906437, respectively.

### 2.4. Transmission Electron Microscopy (TEM)

To analyze the action of antibiotics on* K. pneumoniae* isolates, these isolates were subjected to different sub-MICs of ampicillin, amoxicillin, ceftazidime, cefotaxime, aztreonam, and imipenem (Sigma-Aldrich) at 37°C for 6 hours, according to the origin and susceptibility profile of each isolate, using clinically relevant concentrations (Tables 1 and 2) [25, 26]. In all the processes, a control for the isolate was included under the same conditions and at the same dilutions, without the presence of the antibiotic. After growth, the bacterial cells were centrifuged and fixed using 2.5% glutaraldehyde and 4% paraformaldehyde (Sigma-Aldrich). The cells were postfixed in 1% osmium tetroxide and then contrasted in 5% uranyl acetate (Electron Microscopy Science). Dehydration was carried out using acetone (Sigma-Aldrich) followed by infiltration and embedment of the material in epon 812 resin (Electron Microscopy Science). The samples were viewed under a transmission electron microscope (Zeiss EM109).

### 2.5. Scanning Electron Microscopy (SEM)

The* K. pneumoniae* isolates were subjected to antimicrobial agents using the concentrations and criteria described above (Tables 1 and 2). After growth, the bacterial cells were gently centrifuged and fixed using 2.5% glutaraldehyde (Sigma-Aldrich). Postfixation was performed using 1% osmium tetroxide (Electron Microscopy Science) followed by dehydration using ethanol (Sigma-Aldrich). After dehydration, the material was dried in preparation for metallization and viewing of the bacterial cells under a scanning electron microscope (JEOL JSM-5600 LV).

## 3. Results

### 3.1. Antimicrobial Susceptibility

The susceptibility profiles of the K3C, K16R, K21.1-F, and K58.1-F isolates had previously been determined in other studies [9, 17] and are described in Tables 1 and 2. The* K. pneumoniae* K652 isolate showed resistance to all the antibiotics tested using the VITEK 2 automated system (ampicillin, ampicillin/sulbactam, aztreonam, cephalosporins, cefepime, cefotaxime, cefoxitin, ceftazidime, ciprofloxacin, ertapenem, gentamicin, imipenem, meropenem, and piperacillin/tazobactam), except for amikacin and tigecycline. The macrodilution broth test confirmed the resistance of this isolate to cefotaxime, ceftazidime, and imipenem, with MICs of 256 *μ*g mL^−1^.

### 3.2. Presence of* bla*_SHV_,* bla*_TEM_, and* bla*_KPC_ Genes

The PCR analyses identified the presence of *bla*_TEM_ gene in the three clinical isolates of* K. pneumoniae* and in the K21.1-F isolate from the microbiota. Additionally, the *bla*_SHV_ and *bla*_KPC_ genes were also identified by means of PCR in the* K. pneumoniae* isolate K652.

### 3.3. Sequencing of the* bla*_TEM_ and* bla*_KPC_ Genes

The *bla*_TEM-1_ gene was identified in the* K. pneumoniae* isolate K16R, which also had the *bla*_CTX-M2_ gene [17]. The *bla*_TEM_ gene found in the K3C isolate showed a nucleotide change in relation to the *bla*_TEM-1_ gene at positions 512 G→A and 914 G→A, leading to replacement of Glu-104→Lys and Gly-238→Ser. The *bla*_KPC-2_ gene was identified in the* K. pneumoniae* isolate K652.

### 3.4. Ultrastructural and Morphological Analyses

**Figure 1:**
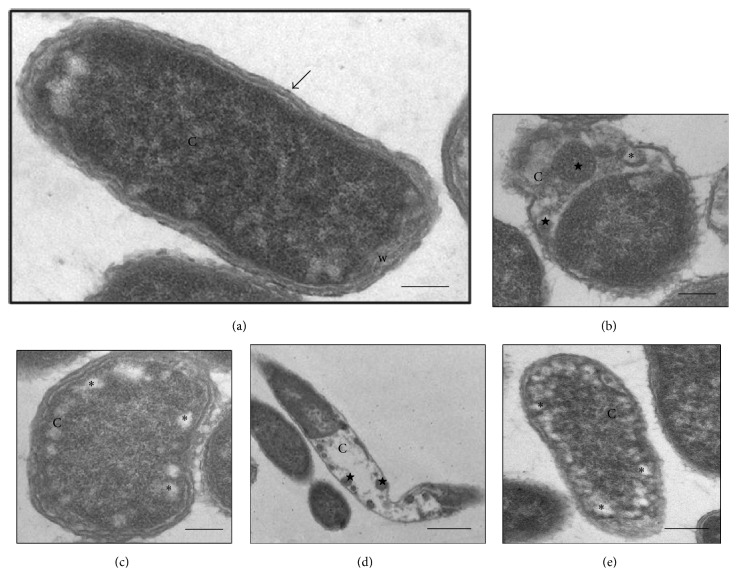
(a–e) Transmission electron micrographs of isolate K3C from* K. pneumoniae*. (a) bacterial cell without treatment (control)—preserved morphology, cytoplasmic membrane (arrow), cell wall (w), and cytoplasm (C) intact. (b-c) Cells subjected to CTX (64 *μ*g mL^−1^)—decreased cytoplasmic material (C), presence of membrane compartments (stars), electron-lucent spaces (asterisks) and disorganization of the membrane and cell wall. (d) Cell subjected to CAZ (32 *μ*g mL^−1^)—elongated aspect morphology with loss of cytoplasmic material (C) and the presence of small membrane compartments (stars). (e) Cell subjected to ATM (32 *μ*g mL^−1^)—normal morphology in the presence of electron-lucent spaces (asterisks) in the cytoplasm (C). Bars = 0.5 *μ*m. CAZ: ceftazidime; CTX: cefotaxime; and ATM: aztreonam.

**Figure 2:**
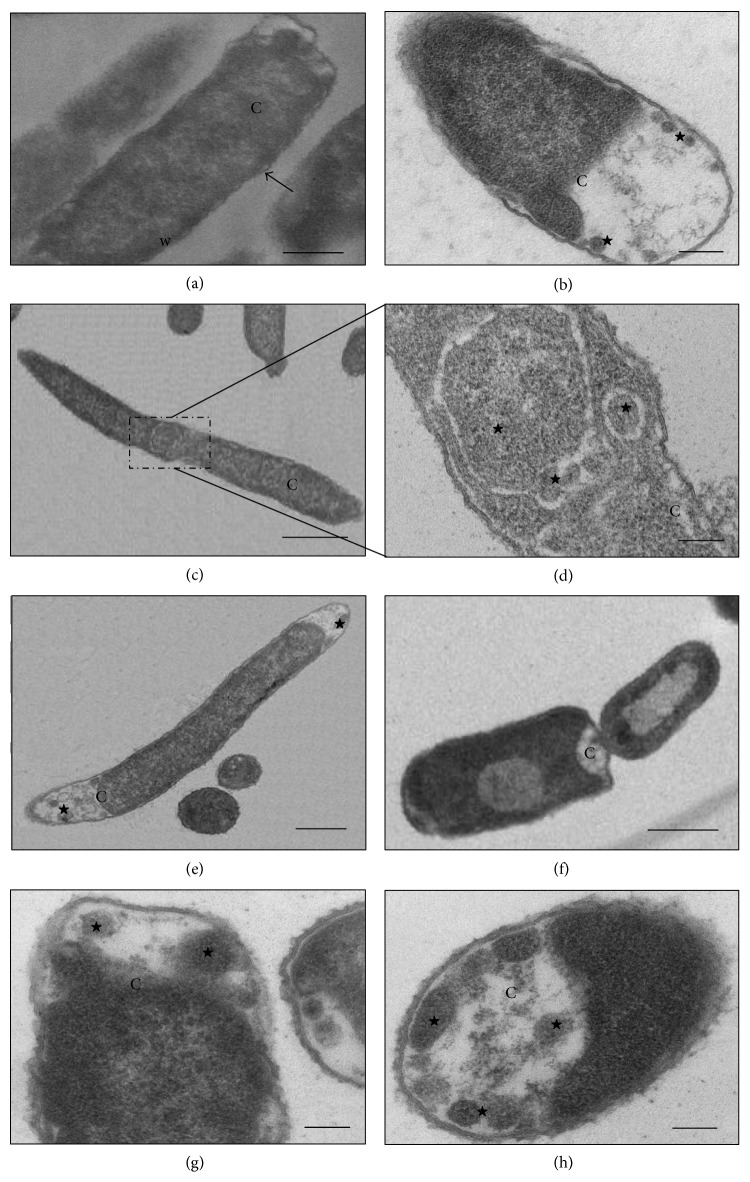
(a–h) Transmission electron micrographs of isolate K16R from* K. pneumoniae*. (a) Untreated bacterial cell—preserved morphology, cytoplasmic membrane (arrow), cell wall (w), and cytoplasm (C) intact. (b) Cell subjected to CTX (64 *μ*g mL^−1^)—presence of large electron-lucent space due to increased periplasmic space (stars) and reduced cytoplasmic material (C). (c–e) Cells subjected to CAZ (16 *μ*g mL^−1^). (c-d) Filamentous cells and the presence of membrane compartments containing cytoplasmic material (star). (e) Elongated cell showing loss of cytoplasmic material (star). (f–h) Cell subjected to ATM (4 *μ*g mL^−1^)—loss of cytoplasmic material (C) and presence of membrane compartments (stars). Bars = 0.5 *μ*m. CAZ: ceftazidime; CTX: cefotaxime; and ATM: aztreonam.

**Figure 3:**
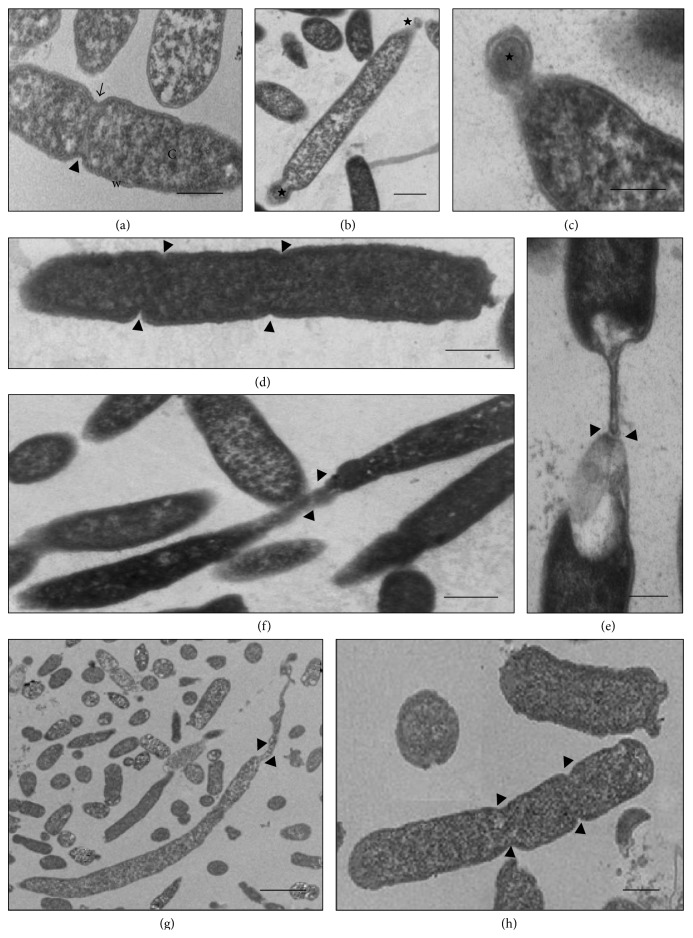
(a–h) Transmission electron micrographs of isolate K652 of* K. pneumoniae*. (a) Untreated bacterial cell—cell wall with preserved morphology (w) and cytoplasm (C) intact, besides regular septation (arrowheads). (b-c) Cell subjected to CTX (98 *μ*g mL^−1^)—presence of cells not grown and rounded (stars) at the ends of cells with normal morphology. (d–f) Cells subjected to CAZ (27 *μ*g mL^−1^)—consecutive formation of septa (arrowheads) providing elongation of bacterial morphology. Observe disorganization of membrane and cell wall with elastic aspect. (g-h) Cell subjected to IMP (16 *μ*g mL^−1^)—morphology of elongated appearance with formation of consecutive septa (arrowheads). Bars = 0.5 *μ*m. CAZ: ceftazidime; CTX: cefotaxime; and IMP: imipenem.

**Figure 4:**
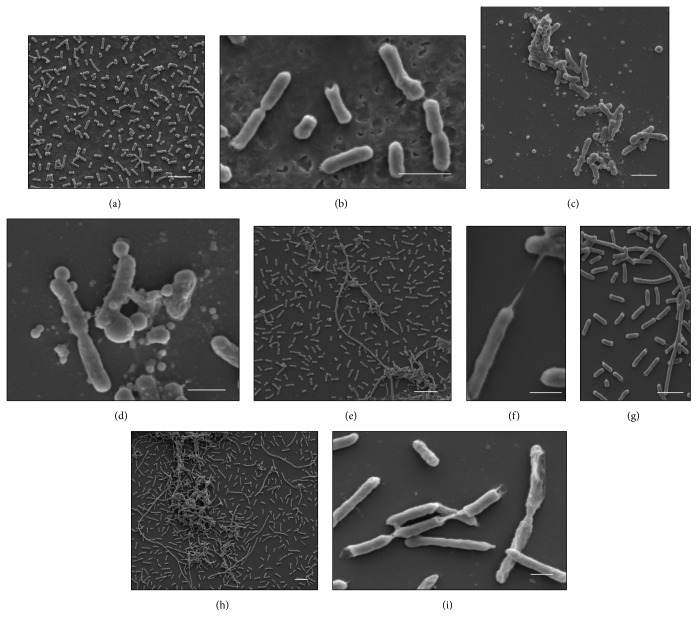
(a–i) Scanning electron micrographs of isolate K652. (a-b) Control cells with preserved morphology. (c-d) Cells subjected to CTX (98 *μ*g mL^−1^). Observe spherical cells that are not grown being formed from cells with preserved morphology. (e–g) Cells subjected to CAZ (27 *μ*g mL^−1^). Observe elongated cells forming bacterial filaments. (h-i) Cells subjected to IMP (16 *μ*g mL^−1^). Observe elongated cells due to unfinished successive divisions. Bars = 2 *μ*m. CAZ: ceftazidime; CTX: cefotaxime; and IMP: imipenem.

**Figure 5:**
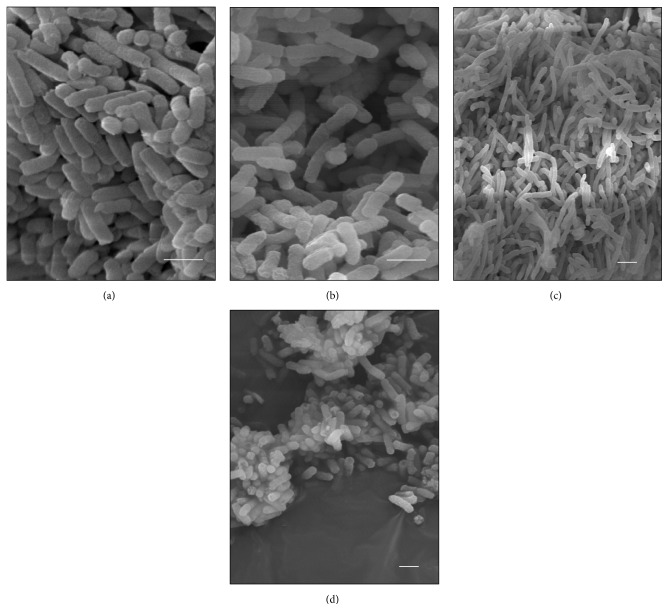
(a–d) Scanning electron micrographs of isolate K16R. (a) Control cells with preserved morphology. (b) Cells subjected to CTX (32 *μ*g mL^−1^). (c) Cells subjected to CAZ (16 *μ*g mL^−1^). (d) Cells subjected to ATM (4 *μ*g mL^−1^). Bars = 2 *μ*m. CAZ: ceftazidime; CTX: cefotaxime; and ATM: aztreonam.

**Figure 6:**
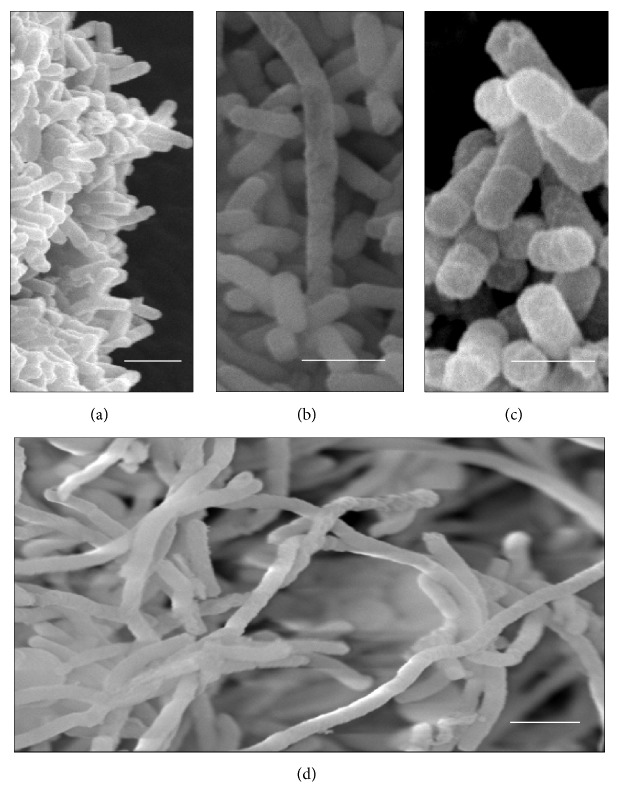
(a–d) Scanning electron micrographs of isolate K3C. (a) Control cells with preserved morphology. (b) Cells subjected to CTX (32 *μ*g mL^−1^). (c) Cells subjected to CAZ (32 *μ*g mL^−1^). (d) Cells subjected to ATM (32 *μ*g mL^−1^). Bars = 2 *μ*m. CAZ: ceftazidime; CTX: cefotaxime; and ATM: aztreonam.

The cells of the control* K. pneumoniae* isolates, which were analyzed without having been subjected to *β*-lactam antibiotics, showed morphology that had been preserved, with an intact cell wall and cytoplasmic contents of electron-dense appearance on analysis by means of TEM. The presence of ribosomes and genetic material distributed in the bacterial cytoplasm could be seen. The cells undergoing a division process showed formation of septa without irregularities (Figures 1(a), 2(a), and 3(a)). The SEM analysis showed conserved rod-shaped bacterial morphology and an average cell length of 1.5 to 3 *μ*m (Figures 4(a), 4(b), 5(a), and 6(a)).

### 3.5. K16R Isolate

Although the K16R isolate presented resistance to cefotaxime, ceftazidime, and aztreonam and had the *bla*_SHV-1_, *bla*_TEM-1_, and *bla*_CTX-M2_ genes, subjection of this isolate to sub-MICs of these antimicrobials caused cellular events that differed from those of the control cells. After subjection to cefotaxime at concentrations of 32 and 64 *μ*g mL^−1^, cells with increased periplasmic space over their entire extent with greater amounts of space at their ends were observed, thus suggesting that the cytoplasm had decreased in volume or retracted (Figure 2(b)). No significant morphological changes to cell size were observed in the SEM analysis (Figure 5(b)).

Subjection of the K16R isolate to ceftazidime at concentrations of 8 and 16 *μ*g mL^−1^ allowed identification of morphological and cellular organizational changes by means of TEM and SEM, in which several elongated cells were viewed. Through TEM, it could be seen that this event occurred because of unfinished cell division, and this was identified through the presence of several consecutive division septa that were incomplete. Some cells also showed membrane cell spaces inside the bacterial cytoplasm and cytoplasmic content identical to the rest of the cell, with ribosomes and genetic material equally distributed (Figures 2(c)–2(e)). This suggests that cell division did not happen, thus enabling formation of membrane compartments within the bacterial cells. Structural disorganization of the continuity of the cytoplasmic membrane of some cells and decreased amounts of bacterial cytoplasm could also be observed. Through SEM, it was possible to identify cells that were more than 15 *μ*m in length (Figure 5(c)).

After the K16R isolate was subjected to application of 4 *μ*g mL^−1^ of aztreonam, some cells presented large electron-lucent spaces in the bacterial cytoplasm with decreased cytoplasmic mass (Figures 2(f)–2(h)). No morphological or cell size changes were identified through SEM (Figure 5(d)).

### 3.6. K3C Isolate

The* K. pneumoniae* isolate K3C also has phenotypic resistance to cefotaxime, ceftazidime, and aztreonam and has the *bla*_SHV-11_ and *bla*_TEM-15_ genes. After this isolate was subjected to 32 and 64 *μ*g mL^−1^ of cefotaxime, some cells showed a decrease in cytoplasmic material with formation of membrane compartments within the cells, which presented internal material containing ribosomes. These cells showed cytoplasmic membrane disorganization. Several cells also contained in their cytoplasm a large number of nonmembrane electron-lucent structures, near the cell membrane, at both tested concentrations (Figures 1(b) and 1(c)). No morphological change was identified through SEM (Figure 6(b)).

Various filamentous cells were seen through TEM and SEM after the K3C isolate was subjected to 16 and 32 *μ*g mL^−1^ of ceftazidime. Through TEM, it could be seen that cell elongation was followed by a decrease in cytoplasmic material in the middle of the dividing cell or at the ends of cells that had already separated. The presence of a large electron-lucent space between the cytoplasmic membrane and cell wall of these cells highlighted the presence of cell compartments separated by membranes that contained cytoplasmic material (Figure 1(d)). Through SEM, cells of up to 18 *μ*m in length could be seen (Figure 6(c)).

The cellular events induced by aztreonam in this isolate, at concentrations of 16 and 32 *μ*g mL^−1^, were discreet. Some cells showed large numbers of nonmembrane electron-lucent spaces in the bacterial cytoplasm, similar to those found after treatment with cefotaxime (Figure 1(e)). However, most of the cells showed conservation of morphology with preservation of the cytoplasmic membrane and cell wall. No morphological changes were observed through SEM (Figure 6(d)).

### 3.7. K652 Isolate

The K652 isolate of* K. pneumoniae* has the *bla*_KPC-2_ gene and shows high phenotypic resistance to carbapenems and third-generation cephalosporins. Subjection of this isolate to both ceftazidime at 27 *μ*g mL^−1^ and imipenem at 16 *μ*g mL^−1^ caused cell filamentation, as seen in analyses using SEM. Cells of more than 50 mm in length could be seen (Figures 4(e)–4(i)). The number of elongated cells seemed to be greater after subjection to imipenem than to ceftazidime (Figures 3(d)–3(h)). The TEM analysis on the K652 isolate subjected to these antibiotics showed that most of the cells showed formation of consecutive division septa, without completing the division process. Many cells also showed elongation and disorganization of the cytoplasmic membrane and cell wall at the site of cell division, thus providing a resilient appearance to these cellular components.

Through SEM, various rod-shaped bacilli that gave rise to nondeveloped cells in the form of cocci could be observed after the K652 isolate was subjected to 98 *μ*g mL^−1^ of cefotaxime (Figures 4(c) and 4(d)). The TEM confirmed that some spherical cells were being formed from rod-shaped cells through changes to the density of the bacterial cytoplasm (Figures 3(b) and 3(c)).

### 3.8. K58.1-F and K21.1-F Isolates

The results from the TEM and SEM analyses on the* K. pneumoniae* isolates from the microbiota, after subjection to amoxicillin and ampicillin, were quite similar to those from the isolates obtained from the hospital environment when the latter were subjected to cefotaxime and aztreonam.

**Figure 7:**
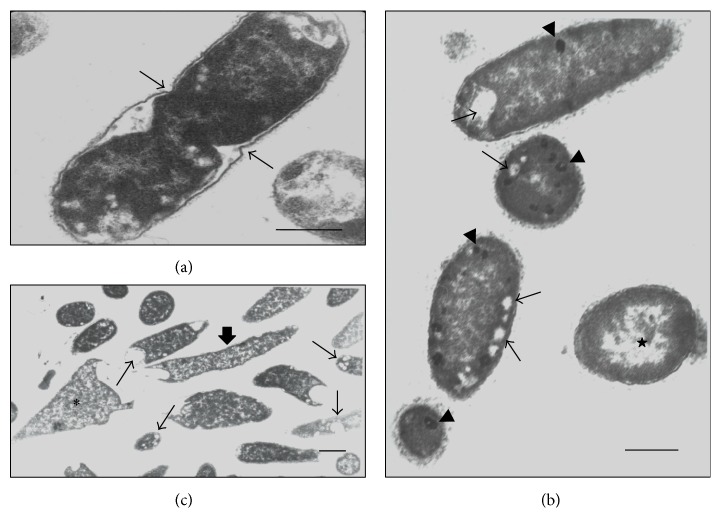
(a–c) Transmission electron micrographs of isolate K21.1-F and K58.1-F of* K. pneumoniae*. (a) Isolate K21.1-F submitted to 0.5 *μ*g mL^−1^ ampicillin. Observe irregular formation of septa in a dividing cell (arrow). (b) Isolate K58.1-F subjected to 0.5 *μ*g mL^−1^ of ampicillin. Cells present electron-lucent (fine arrow), and electron-dense spaces with different sizes and shape throughout the bacterial cytoplasm (arrowheads), in addition to reduction of cytoplasmic contents (star). (c) Isolate K58.1-F subjected to 4 *μ*g mL^−1^ of amoxicillin. The cells exhibit altered morphologies with cell elongation (large arrow) and undefined forms (asterisk), with the presence of electron-lucent structures with shape and varying sizes scattered throughout the cell cytoplasm (fine arrow). Bars = 0.5 *μ*m.

The K58.1-F isolate showed phenotypic resistance to amoxicillin and intermediate resistance to ampicillin, when exposed to sub-MICs of these antibiotics (Table 2). This was demonstrated by the enlarged electron-lucent spaces at the ends of the cells, in comparison with the control, as seen through TEM, thus suggesting that there was a decrease in the cytoplasmic content. Large quantities of electron-lucent spaces and nonmembrane electron-dense structures were also identified in the bacterial cytoplasm of several cells (Figures 7(b) and 7(c)). These cellular events became more pronounced with higher concentrations of each drug, thus demonstrating that there was a dose-dependent correlation.

Although the K21.1-F isolate showed higher MICs for amoxicillin and ampicillin than that of the K58.1-F isolate, the TEM analysis after the isolates were subjected to sub-MICs of these antibiotics showed that the results from the two isolates were similar. Large quantities of electron-lucent spaces and nonmembrane electron-dense structures were identified in the bacterial cytoplasm of several cells of the K21.1-F isolate, even at the lowest concentration tested (0.5 *μ*g mL^−1^) (Figure 7(a)). Several cells with irregular formation of septa during bacterial division, but without loss of cytoplasmic content even at higher concentrations, were simultaneously observed (Figure 7). The SEM analysis did not show any significant changes to any of the antibiotic concentrations tested, in relation to the K21.1-F and K58.1-F isolates.

## 4. Discussion

Production of *β*-lactamases is one of the main mechanisms of resistance among isolates of the Enterobacteriaceae family. ESBL and KPC in particular are often found in* K. pneumoniae* clinical isolates, thus conferring a phenotype that is resistant to the main *β*-lactams used in clinical medicine.

The *bla*_TEM-15_, *bla*_CTX-M2_, and *bla*_KPC-2_ genes, found, respectively, in the K3C, K16R, and K652 isolates, encode enzymes of ESBL and carbapenemase type. These genes have previously been reported in different bacterial species [27–29]. The changes of Glu→Lys at position 104 and Gly→Ser at position 238 of the *bla*_TEM-15_ gene are known to favor increased substrate fixation and an enzyme-active site, thus giving rise to an enzyme with increased affinity and catalytic efficiency for oxyiminocephalosporins [29]. ESBL CTX-M belonging to the CTX-M2 phylogenetic group is one of the CTX-M-type enzymes most found in Enterobacteriaceae isolates in Brazil [28], with high hydrolysis power compared with third-generation cephalosporins, especially cefotaxime.

KPC *β*-lactamases efficiently hydrolyze penicillins, cephalosporins, carbapenems, and aztreonam and are inhibited by clavulanic acid and tazobactam [28]. The K652 isolate presents the resistance gene *bla*_KPC-2_. This gene was first detected in Brazil in* K. pneumoniae* isolates obtained from patients hospitalized in Recife, PE [27]. Since then, other studies have demonstrated the presence of this gene in* K. pneumoniae* isolates obtained from several other states in Brazil [27, 28].

Cases of infections by isolates producing these enzymes give rise to considerable concern regarding the most appropriate antibiotic therapy. This is because bacterial growth often does not change, and cell death due to subjection to *β*-lactams does not occur. In this context, the present study was designed to demonstrate the* in vitro* effect of imipenem, penicillin, third-generation cephalosporins and aztreonam on MDR* K. pneumoniae*, and microbiota isolates obtained from individuals under different clinical conditions. This variability made it possible to analyze the effects of *β*-lactam antibiotics in isolates with distinct genetic and phenotypic resistance profiles, which would have the capacity to evolve differently in their host organisms.

Subjection of the K3C, K16R, and K652 isolates to different concentrations of ceftazidime, and of the K652 isolate to imipenem, caused cell filamentation in all the isolates analyzed, with formation of several consecutive septa and disorganization of the cell membrane and cell wall in several cells. This induction of cell filamentation was previously reported in other studies using sensitive and resistant isolates from different bacterial species, including* Serratia marcescens*,* K. pneumoniae*, and* P. aeruginosa* subjected to third-generation cephalosporins, carbapenems, and monobactams. These studies showed that inactivation of different penicillin-binding proteins may be associated with inability to complete the cell division process after replication of the bacterial mass [4, 5, 16, 30].

In our study, the presence of bacterial filaments and cell disorganization caused by* K. pneumoniae* isolates after subjection to ceftazidime and imipenem makes us believe that these antibiotics have some residual activity, despite the presence of genes that code for ESBL and KPC. However, even though the *β*-lactam antibiotics tested here were capable of causing changes to the* K. pneumoniae* isolates used in this study, these antibiotics were clearly incapable of interfering with the survival of isolates with a resistant phenotype.

Because of the great need for effective therapeutic options for treating MDR bacterial isolates, new therapeutic combinations have been tested. Hirsch et al. conducted a study that showed that meropenem and amikacin in combination were able to inhibit* in vitro* and* in vivo* growth of resistant* K. pneumoniae* isolates that produced KPC-2 and KPC-3 [15]. In the present study, it was found that imipenem and ceftazidime alone were able to promote* in vitro* cellular changes that included loss of cytoplasmic material, filamentation, and disorganization of the cell membrane and cell wall at the division site. Understanding the effect of antibiotics alone on MDR* K. pneumoniae* isolates is extremely important because this enables understanding of how the development of therapeutic combinations can effectively contribute towards treating the infections caused by these isolates. In our study, resistant* K. pneumoniae* isolates subjected to clinically relevant concentrations *β*-lactam antibiotics seemed to have responses similar to those of sensitive cells at low concentrations of these antibiotics.

Deloney and Schiller [31] used* Helicobacter pylori*-sensitive isolates subjected to sub-MICs of *β*-lactam antibiotics, including ampicillin and aztreonam, and showed that the bacterial cells became filamentous when subjected to aztreonam and spherical when subjected to other *β*-lactams. Our data showed that aztreonam was unable to induce cell filamentation in the clinical isolates tested. However, the appearance of electron-lucent spaces suggested that loss or retraction of cytoplasmic contents occurred, in the same way that occurred when microbial isolates were subjected to ampicillin and amoxicillin.

Our results demonstrate that* K. pneumoniae* isolates harboring different genes that encode for *β*-lactamases can undergo ultrastructural changes when subjected to sub-MICs of *β*-lactam drugs, thus suggesting that this antimicrobials have residual activity* in vitro*, despite the phenotypic resistance presented in the isolates analyzed.

## Figures and Tables

**Table 1:** MICs of *K*. *pneumoniae* isolates obtained in hospital and sub-MICs used for analysis by electron microscopy.

Isolates	Cefotaxime			Ceftazidime			Aztreonam		
	(*μ*g mL^−1^)			(*μ*g mL^−1^)			(*μ*g mL^−1^)		
ESBL	MICs^a^	Sub-MICs^b^		MICs^a^	Sub-MICs^b^		MICs^a^	Sub-MICs^b^	
		TEM^c^	SEM^d^		TEM^c^	SEM^d^		TEM^c^	SEM^d^
K3C	>256	64; 32	32	128	32; 16	32	64	32; 16	32
K16R	>256	64; 32	32	32	16; 8	16	16	4	4

Isolates	Cefotaxime			Ceftazidime			Imipenem		
	(*μ*g mL^−1^)			(*μ*g mL^−1^)			(*μ*g mL^−1^)		
KPC	MICs^a^	Sub-MICs^b^		MICs^a^	Sub-MICs^b^		MICs^a^	Sub-MICs^b^	
		TEM^c^	SEM^d^		TEM^c^	SEM^d^		TEM^c^	SEM^d^

K652	≥256	98	98	≥256	27	27	≥128	16	16

^a^MICs: minimum inhibitory concentrations; ^b^sub-MICs: sub-minimum inhibitory concentrations; ^c^TEM: transmission electron microscopy; and ^d^SEM: scanning electron microscopy.

**Table 2:** MICs of *K*. *pneumoniae* isolates obtained of microbiota and sub-MICs used for analysis by electron microscopy.

Isolates	Ampicillin (*μ*g mL^−1^)			Amoxicillin (*μ*g mL^−1^)		
	MICs^a^	Sub-MICs^b^		MICs^a^	Sub-MICs^b^	
		TEM^c^	SEM^d^		TEM^c^	SEM^d^
K58.1-F	16	0.5; 2; 8	0.5; 8	32	0.5; 4; 16	0.5; 16
K21.1-F	128	0.5; 16; 32; 64	16; 32; 64	>128	0.5; 4; 16; 64; 128	0.5; 16; 128

^a^MICs: minimum inhibitory concentrations; ^b^sub-MICs: sub-minimum inhibitory concentrations; ^c^TEM: transmission electron microscopy; and ^d^SEM: scanning electron microscopy.
